# Spatiotemporal imaging of immune dynamics: rethinking drug efficacy evaluation in cancer immunotherapy

**DOI:** 10.3389/fimmu.2025.1609606

**Published:** 2025-05-16

**Authors:** Xiaowei Zhang, Cheng Cheng, Xiaoyang Qu, Pengyun Wang, Dawei Zhang, Shuhong Dai

**Affiliations:** ^1^ Department of Orthopedics, Zibo Central Hospital, Zibo, China; ^2^ Department of Cardiology, Zibo Central Hospital, Zibo, China; ^3^ Department of Infectious Diseases, Zibo Central Hospital, Zibo, China

**Keywords:** tumor immune microenvironment, spatiotemporal imaging, immunotherapy evaluation, in vivo imaging, multiplexed tissue imaging

## 1 Introduction: the need for a new paradigm in drug efficacy evaluation

The evaluation of drug efficacy remains a central pillar in the development and clinical application of immunotherapies. Traditionally, therapeutic success has been assessed using static and endpoint-based biomarkers, such as tumor size reduction, survival extension, or changes in a limited set of immune markers (e.g., PD-L1 expression or circulating cytokine levels) (1–3). While these metrics have guided many clinical decisions, they often fail to capture the full complexity and heterogeneity of dynamic immune responses, particularly in the context of immuno-oncology, where therapeutic effects can be delayed, indirect, or spatially restricted (4).

Recent advances in immunotherapy, including immune checkpoint inhibitors, CAR-T cells, and tumor vaccines, demand a new framework for evaluating drug efficacy—one that accounts for the spatiotemporal dynamics of immune responses (5, 6). Immune cells may transiently infiltrate tumors, reorganize spatially, or engage in local interactions that are critical for therapeutic outcomes but remain undetectable using conventional assays. Conventional assays typically employed for immune response assessment include IHC, enzyme-linked immunosorbent assay (ELISA), flow cytometry, and quantitative polymerase chain reaction (qPCR). Although informative, these assays provide endpoint measurements and generally lack the resolution to detect transient, spatially restricted, or dynamic interactions of immune cells within the tumor microenvironment (TME) (4). The immune landscape is not static, and responses can evolve rapidly over time and vary widely between tumor regions (7).

In this evolving therapeutic landscape, emerging imaging technologies—ranging from multiplexed spatial imaging at the tissue level to real-time *in vivo* imaging platforms—offer a transformative opportunity (6). Emerging imaging technologies that are significantly enhancing our understanding of the tumor-immune microenvironment include Multiplexed Ion Beam Imaging (MIBI), Imaging Mass Cytometry (IMC), Cyclic Immunofluorescence (CycIF), CO-Detection by Indexing (CODEX), Positron Emission Tomography (PET), Single-Photon Emission Computed Tomography (SPECT), Magnetic Resonance Imaging (MRI), and Intravital Microscopy. These advanced methods provide detailed spatial, temporal, and molecular resolution, enabling visualization of immune cell dynamics and interactions within the tumor microenvironment at levels previously unattainable by conventional assays (4, 8, 9). These tools allow researchers and clinicians to visualize immune activity where it happens and as it unfolds. By directly observing how drugs engage their targets, modulate the immune microenvironment, and impact immune cell behavior, imaging can provide a richer and more accurate representation of therapeutic efficacy (9).

This opinion article argues that drug efficacy evaluation must shift beyond static biomarkers toward integrated, image-guided approaches that combine spatial, temporal, and functional insights. Such a paradigm shift could greatly enhance precision medicine and improve therapeutic outcomes in immunotherapy.

## 2 The rise of multiplex and spatial imaging for tissue-level analysis

Tissue-level drug efficacy evaluation has historically relied on basic histological techniques and immunohistochemistry (IHC), offering limited information on the complex spatial relationships that define immune response (10). While traditional biomarkers like PD-L1 or CD8^+^ T cell counts remain clinically relevant, they offer a static and often incomplete snapshot (11). As our understanding of tumor–immune dynamics deepens, the ability to analyze immune responses in spatial context has become indispensable.

**Figure 1:**
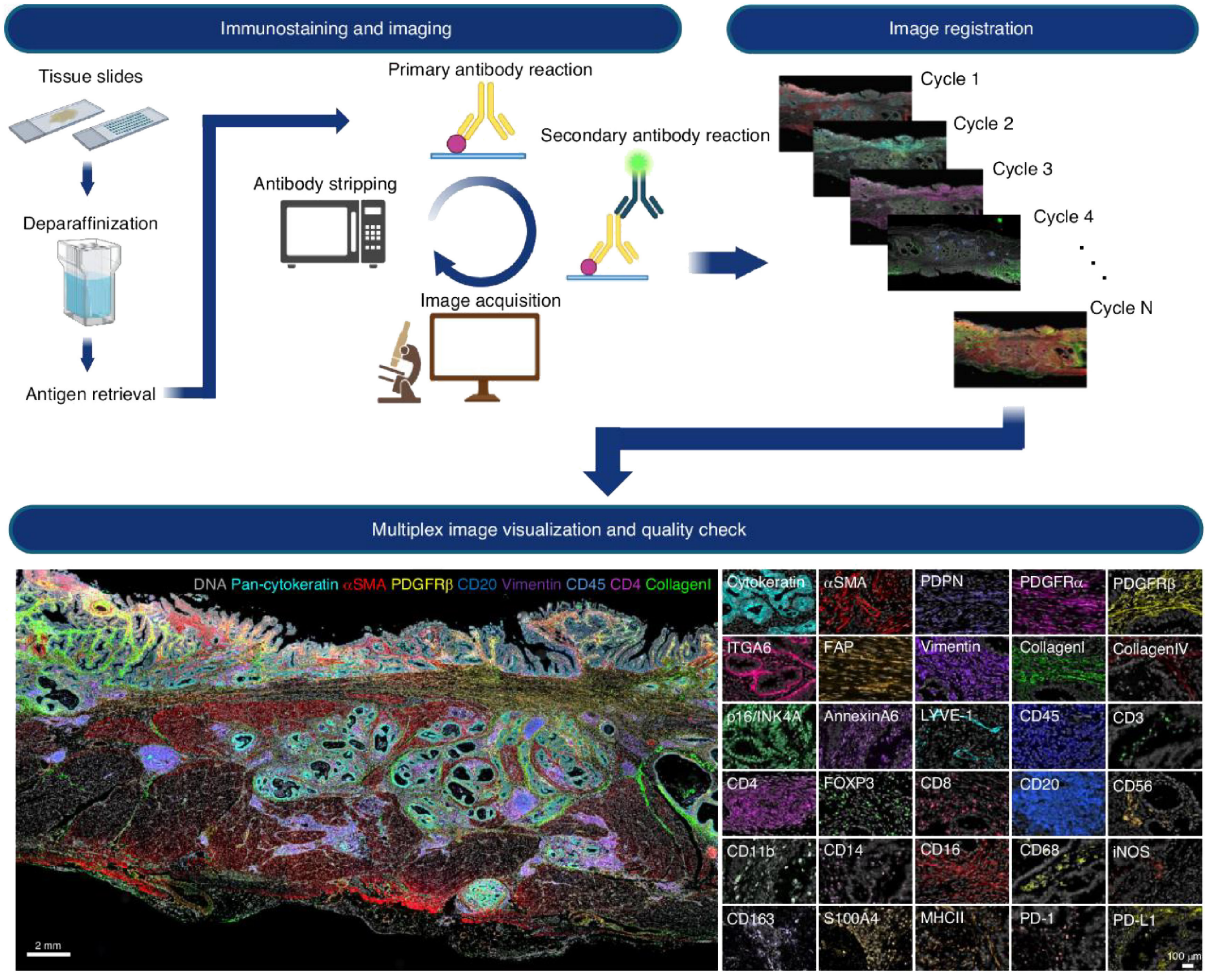
Cyclic multiplex immunofluorescence imaging workflow and representative results. Top panel: Iterative imaging process begins with formalin-fixed paraffin-embedded (FFPE) tissue slides undergoing deparaffinization and antigen retrieval. Each cycle consists of primary and secondary antibody labeling, image acquisition, and fluorophore stripping. The resulting images from multiple cycles are computationally aligned (registered) to generate a spatially resolved dataset containing dozens of protein markers. Bottom panel: Composite visualization of a multiplex image from tumor tissue, displaying color-coded spatial expression of markers such as DNA (blue), pan-cytokeratin (cyan), αSMA (red), PDGFRβ (green), CD20, CD45, CD4, CD8, vimentin, and collagen. The right side presents selected single-marker images (e.g., ITGA6, CD3, CD8, MHC-II, PD-L1), enabling detailed analysis of immune cell subsets and structural compartments within the tumor microenvironment. Reproduced with permission from ref (4).

Multiplexed spatial imaging technologies have emerged as powerful tools to overcome these limitations (8). Techniques such as Imaging Mass Cytometry (IMC), Multiplexed Ion Beam Imaging (MIBI), Cyclic Immunofluorescence (CycIF), and CO-Detection by Indexing (CODEX) allow simultaneous visualization of 30 to over 60 proteins within intact tissue sections, preserving spatial architecture (12–14). The multiplex spatial imaging pipeline is illustrated in **Figure 1**, which includes both the experimental workflow (top panel) and a representative imaging output (bottom panel). The workflow begins with tissue preparation steps including paraffin removal and antigen retrieval, followed by iterative rounds of antibody staining and image acquisition (4). Each cycle involves the application of a primary antibody and a fluorescently labeled secondary antibody, after which the tissue is imaged and the signal is chemically stripped. This process is repeated multiple times (Cycle 1 to Cycle N), each targeting a distinct set of protein markers.

Once imaging is complete, all cycle images are computationally registered to produce a high-dimensional, spatially resolved composite image. The bottom panel shows an example of such a multiplex image, highlighting distinct cell types and structures within the TME. Markers such as DNA, pan-cytokeratin, αSMA, PDGFRβ, CD20, CD45, CD4, CD8, vimentin, and collagen reveal a rich tissue architecture that includes epithelial structures, stromal fibroblasts, immune infiltrates, and extracellular matrix components. Additional single-channel panels demonstrate high-resolution staining of over 20 individual markers, facilitating the detailed classification of immune and stromal subtypes and their spatial distribution.

Building upon workflows like the one shown in **Figure 1**, these technologies offer unprecedented resolution into the tumor immune microenvironment (TIME), revealing how immune cells are distributed relative to tumor cells, vasculature, and each other. For example, the presence of tertiary lymphoid structures (TLS), spatial clustering of CD8+ T cells near tumor nests, or exclusion of effector T cells from tumor cores are spatial features that have all been correlated with response or resistance to immune checkpoint inhibitors (15). Such findings underscore the need to incorporate spatial biomarkers into drug evaluation pipelines. Moreover, spatial imaging enables retrospective evaluation of clinical trial specimens, helping explain heterogeneous responses. In trials where traditional biomarkers fail to predict outcomes, spatial immune phenotypes—such as myeloid-rich immunosuppressive niches or immune deserts—can offer mechanistic insights and support patient stratification strategies (16). Emerging applications also include the study of therapeutic interventions themselves, such as evaluating immune infiltration post-vaccination or CAR-T cell localization after infusion. Multiplex imaging allows researchers to quantify how drugs reshape the TIME, revealing shifts in cell phenotypes, activation states, or the emergence of suppressive cell types (17).

Despite their promise, integration of these technologies into clinical workflows remains limited due to high cost, labor intensity, and analytical complexity. Nonetheless, advances in automation, cloud-based analysis platforms, and machine learning-driven interpretation are making spatial imaging increasingly accessible. When paired with clinical endpoints, spatial imaging offers not only correlative insights but the potential for spatial biomarker-driven patient selection and real-time therapy monitoring.

In sum, multiplex and spatial imaging have redefined how immune activity within tissues can be visualized, quantified, and interpreted. They offer a much-needed bridge between molecular data and tissue-level functional context, laying the foundation for more nuanced and effective immunotherapy evaluation.

## 3 *In vivo* and real-time imaging: capturing immune dynamics beyond the slide

​The evaluation of drug efficacy in immunotherapy has traditionally relied on static biomarkers and endpoint assessments, which often fail to capture the dynamic nature of immune responses (18). Recent advancements in *in vivo* and real-time imaging technologies have revolutionized our ability to monitor immune dynamics, providing deeper insights into therapeutic mechanisms and facilitating the development of more effective treatment strategies (19, 20). ​

### 3.1 Advancements in *in vivo* and real-time imaging technologies

*In vivo* imaging techniques have evolved to allow non-invasive visualization of immune cells within their native environments, enabling the study of cellular behaviors and interactions over time (21, 22). **Table 1** provides an overview of key imaging modalities employed in immunotherapy research, highlighting their spatial and temporal resolution, primary applications, as well as their respective advantages and limitations. Key technologies include intravital microscopy, positron emission tomography (PET), single-photon emission computed tomography (SPECT), and magnetic resonance imaging (MRI). These modalities offer unique advantages in tracking immune cell migration, activation, and function in response to immunotherapies. ​

**Table 1:** Key imaging modalities for drug efficacy evaluation in immunotherapy.

Imaging Modality	Spatial Resolution	Temporal Resolution	Key Applications	Strengths	Limitations
CycIF	High (single-cell)	Static (tissue-based)	Immune cell phenotyping, spatial context	High multiplexing; preserved tissue architecture	Labor-intensive; ex vivo only
IMC	High	Static	Deep profiling of TIME	>30 markers simultaneously; metal-tagged antibodies	Costly; limited throughput
PET (e.g., anti-PD-L1 tracer)	Whole-body	Real-time	Monitoring checkpoint expression, cell trafficking	Non-invasive; whole-body coverage	Limited resolution; radiation exposure
Intravital Microscopy	Very high	Real-time	T cell–tumor interactions, vascular dynamics	Direct observation of live processes	Invasive; preclinical only
MRI (e.g., SPIO-labeled cells)	Moderate	Real-time	Immune cell tracking	High soft-tissue contrast; clinical-grade	Low sensitivity for some targets

### 3.2 Intravital microscopy

Intravital microscopy provides high-resolution, real-time visualization of cellular processes in live animals (23). This technique has been instrumental in elucidating the dynamics of T cell infiltration into tumors, mechanisms of cancer cell killing, and the role of myeloid cells in tumor progression. For instance, Intravital microscopy uniquely enables direct visualization of dynamic immune cell behaviors such as T-cell migration patterns, stable versus transient interactions with tumor cells, and their real-time cytotoxic effects *in vivo*. For instance, studies using intravital imaging have illustrated how stable, long-lasting interactions between cytotoxic T lymphocytes (CTLs) and tumor cells correlate with enhanced tumor cell apoptosis, providing mechanistic insights that static assays fail to capture (24, 25). Conversely, transient interactions may indicate ineffective immune responses and tumor evasion strategies (26).

### 3.3 Positron emission tomography and single-photon emission computed tomography

PET and SPECT generate imaging contrast through the use of radiolabeled probes. In PET imaging, positron-emitting isotopes such as ^18^F, ^68^Ga, or ^89^Zr emit positrons upon decay, which interact with electrons to produce gamma photons detectable by the PET scanner. SPECT imaging utilizes gamma-emitting isotopes like ^99^mTc or ^111^In, directly detecting gamma photons via gamma cameras. These radiotracers can be conjugated to antibodies, peptides, or metabolic substrates, enabling the specific visualization of immune cells, tumor markers, or molecular processes non-invasively with high sensitivity (27, 28). PET and SPECT imaging utilize radiolabeled tracers to detect specific molecular targets, offering whole-body insights into immune cell distribution and activity (29). These modalities have been employed to monitor the expression of immune checkpoints, such as PD-1/PD-L1, and to assess the biodistribution of therapeutic antibodies. For example, PET imaging with radiolabeled anti-PD-L1 antibodies has enabled the non-invasive assessment of PD-L1 expression in tumors, providing valuable information for patient stratification and treatment planning (30). ​

### 3.4 Magnetic resonance imaging and cancer vaccine imaging: tracking immune activation *in vivo*

MRI offers high-resolution anatomical imaging with excellent soft-tissue contrast, making it suitable for tracking labeled immune cells *in vivo* (31, 32). Superparamagnetic iron oxide (SPIO) nanoparticles have been used to label various immune cell populations, allowing their migration and accumulation in tumors to be visualized. SPIO nanoparticles label immune cells through *ex vivo* incubation followed by reinfusion or via antibody-mediated targeting of surface markers *in vivo*. Upon administration, SPIO-labeled cells disturb local magnetic fields detectable by MRI, producing contrast enhancement in images. However, the inherent limitation is the passive accumulation of SPIO nanoparticles in tumors via the enhanced permeability and retention (EPR) effect, which may obscure the precise identification of specific immune populations. Recent advances involve coupling SPIO nanoparticles with specific antibodies or ligands to improve targeting specificity, reducing nonspecific tumor uptake, and enhancing cellular resolution in MRI (33, 34). This approach has been applied to monitor the recruitment of cytotoxic T lymphocytes (CTLs) and regulatory T cells (Tregs) following immunotherapy, providing insights into the mechanisms underlying therapeutic responses (35).

Moreover, the efficacy of cancer vaccines relies on the activation and recruitment of antigen-specific T cells to tumor sites (36). *In vivo* imaging has been employed to monitor these processes, providing insights into vaccine-induced immune responses. MRI tracking of SPIO-labeled dendritic cells, used as vaccine adjuvants, has demonstrated successful migration to lymph nodes and subsequent T cell activation, correlating with tumor regression in preclinical models (37). Together, these applications illustrate how MRI serves as a powerful platform to visualize and quantify immune activation triggered by cancer vaccines, complementing conventional biomarker-based evaluation.

### 3.5 Tracking CAR T-cell therapy

Chimeric antigen receptor (CAR) T-cell therapy has shown promise in treating certain hematologic malignancies (38, 39). *In vivo* imaging has been pivotal in tracking the migration, expansion, and persistence of CAR T cells post-infusion. For instance, PET imaging using ^89^Zr-labeled CAR T cells has allowed researchers to monitor the trafficking of these cells to tumor sites, correlating their accumulation with therapeutic outcomes (40). While CAR T cell expansion and persistence can indeed be quantitatively monitored through blood sampling, imaging modalities such as PET, SPECT, or MRI provide complementary insights into spatial biodistribution, trafficking, and infiltration of CAR T cells into solid tumor masses or sanctuary sites not readily accessible via peripheral blood analysis. Imaging approaches thus are invaluable for assessing CAR T cell targeting efficacy, understanding resistance mechanisms, and optimizing therapy regimens, especially in scenarios involving solid tumors or metastatic niches beyond hematologic contexts (41, 42).​

### 3.6 Assessing immune checkpoint inhibitor therapy

Immune checkpoint inhibitors targeting PD-1/PD-L1 and CTLA-4 have revolutionized cancer treatment. *In vivo* imaging has facilitated the evaluation of these therapies by enabling the visualization of dynamic changes in immune cell infiltration and activation within the tumor microenvironment (43). For example, PET imaging with radiolabeled PD-1 antibodies has been used to assess PD-1 expression levels in tumors, aiding in the prediction of patient responses to checkpoint blockade therapies (44).

### 3.7 From technical barriers to imaging innovation

Despite the advancements, several challenges hinder the widespread clinical adoption of *in vivo* immune imaging. Technical limitations, such as the need for highly specific and sensitive imaging agents, and the potential for tracer-induced alterations in cell behavior, must be addressed (45). Additionally, standardization of imaging protocols and data interpretation is essential to ensure reproducibility and comparability across studies (20). ​

Future research should focus on developing novel imaging probes with enhanced specificity for immune cell subsets and activation states. Combining multiple imaging modalities, such as PET/MRI, could leverage the strengths of each technique, providing comprehensive insights into immune dynamics. Furthermore, integrating *in vivo* imaging data with other biomarkers and clinical parameters may enhance predictive models for treatment responses, ultimately guiding personalized immunotherapy strategies (46). ​

In summary, *in vivo* and real-time imaging technologies have significantly advanced our ability to monitor immune dynamics beyond traditional histological methods. By providing spatiotemporal insights into immune responses, these techniques offer valuable tools for evaluating and optimizing immunotherapies, paving the way for more effective and personalized cancer treatments.

## 4 Challenges and integration into clinical practice

Despite the significant promise of advanced imaging technologies in drug efficacy evaluation, several critical challenges continue to limit their widespread adoption in clinical settings. These challenges span technical, operational, analytical, and regulatory domains (47). One of the foremost technical challenges lies in the development and standardization of imaging agents and protocols. Many imaging platforms, especially *in vivo* real-time modalities such as PET, SPECT, and intravital microscopy, rely on customized tracers, labeled antibodies, or nanoparticles that require rigorous validation. These reagents often lack regulatory approval for routine human use and may suffer from variability in synthesis, stability, or immunogenicity (45). Additionally, achieving sufficient resolution, sensitivity, and specificity in a clinical setting—while maintaining patient safety—remains an ongoing challenge, particularly in deep-tissue imaging.

From an operational perspective, the infrastructure required for advanced imaging is substantial. High-end platforms such as imaging mass cytometry, multiplexed ion beam imaging, or hybrid PET/MRI systems are costly to install and maintain. Furthermore, the execution of multi-modal imaging studies demands highly skilled personnel, cross-disciplinary coordination (e.g., pathology, radiology, immunology), and extended processing times, all of which strain hospital resources and reduce scalability. Data analysis and interpretation pose further hurdles. Imaging datasets are large, multidimensional, and complex, requiring bioinformatics expertise, machine learning pipelines, and standardized analytic workflows. Currently, there is a lack of consensus on how to translate spatial or dynamic imaging findings into clinical decisions. While some spatial biomarkers have shown predictive power in trials, few have undergone prospective validation or regulatory qualification as companion diagnostics (48).

To successfully integrate these technologies into clinical immunotherapy practice, several steps are needed. These include the development of standardized imaging protocols, harmonization of analysis tools across platforms, and validation of predictive imaging biomarkers in large, multicenter cohorts. Moreover, regulatory frameworks must evolve to accommodate dynamic and spatial biomarkers, with pathways for the approval of imaging-based diagnostics and clinical decision tools. With strategic investment and collaboration, imaging can shift from an academic asset to a routine pillar of personalized cancer care.

## 5 Outlook and future perspectives

As immunotherapy continues to reshape the oncology landscape, there is a growing consensus that traditional, static methods of drug efficacy assessment are no longer sufficient. The future of immunotherapy evaluation lies in integrating imaging technologies that can provide comprehensive spatial, temporal, and functional information—enabling a more dynamic and nuanced understanding of immune responses at both tissue and whole-body levels. The convergence of tissue-level multiplex imaging and *in vivo* real-time imaging marks a major step forward. Multiplex platforms like CODEX and IMC offer unprecedented granularity in characterizing the tumor immune microenvironment, while non-invasive modalities such as PET, MRI, and intravital microscopy allow longitudinal monitoring of immune activity and therapeutic impact. Together, these technologies offer the potential to build a unified, high-resolution view of drug–immune system interactions that can guide real-time clinical decisions.

Looking ahead, the integration of these platforms with computational tools—particularly artificial intelligence (AI) and machine learning—will be key. These approaches can help process vast, multidimensional datasets to identify predictive patterns, generate response signatures, and even forecast resistance. Additionally, combining imaging data with other omics layers (e.g., genomics, transcriptomics, proteomics) will further enhance our ability to stratify patients and tailor therapies.

To fully realize this potential, future efforts must focus on standardization, scalability, and clinical validation. Imaging protocols should be harmonized across institutions, and regulatory frameworks must evolve to recognize imaging-based spatial and functional biomarkers as legitimate endpoints in clinical trials. Equally important is the development of user-friendly analytical platforms that can democratize access to high-content imaging, even in resource-limited settings. In conclusion, imaging technologies are poised to transition from passive diagnostic tools to active drivers of precision immunotherapy. Their ability to visualize immune dynamics in space and time offers a powerful avenue to improve therapeutic evaluation, optimize patient selection, and ultimately enhance clinical outcomes in cancer immunotherapy.
